# On the Galbanum Plant

**Published:** 1852-10

**Authors:** F. A. Buhse


*On the Galbanum Plant.*—By F. A. Buhse. The author states, that in his travels in Persia he discovered the plant which yields galbanum. In June, 1848, he found it on the declivities of the Demawend. It is a ferula, from the stalks of which a liquid issues abundantly, by the odour and nature of which he immediately recognized galbanum, and his guides assured him, moreover, that galbanum is gathered from this plant. The author has not not yet distinctly determined the plant. It appears to differ from *Ferula erubescens* (*Annales des Sciences*, iii., Ser. 1844, p. 316.) only by the absence of commissural vitæ; but as neither Aucher-Eloy nor Kotschy, who have both collected the Ferula erubescens, make any mention of its yielding galbanum, the author is in doubt whether his plant be the same, or a variety of it. Don's genus Galbanum (Trib. Sibrinæ) and Lindley's Opaïdia (Trib. Smyrneæ) do not agree with the plant seen by Buhse, unless that both of these authors have made their descriptions from imperfect fruits, or that there exist other plants which yield galbanum. The plant which Buhse describes is called, in some parts of Persia, *Khassuch* (not *Kasneh*, which means Cichor intybus, nor Gäshnis, which is Coriand. sativum,) and appears to be confined to certain districts of Persia. In the whole large district of the Elburs-chain, from the south-east angle to the south-west angle of the Caspian Sea, it is only found in the neighbourhood of the Demawend; but here at an elevation of from 4000 to 8000 feet, and even on the declivity of the top of the Demawend. It exists neither on the mountains of Talysch, nor in the districts of Karadah and Tabris. It is said to re-appear on the Mount Alwend, near Hamadan, and in the neighbourhood of the great salt-desert. Near Hamadan, Aucher-Eloy has gathered his Ferula erubescens, and this supports the supposition that the author's plant is the same. In the salt-desert itself Buhse did not meet with it again. The inhabitants of the Demawend collect the gum-resin, which issues spontaneously from the lower part of the stalk; they do not make incisions in the plant; but it is not at this place that the galbanum is collected for commercial purposes. When fresh, the gum-resin is white like milk, liquid, and somewhat glutinous. In the air it soon becomes yellow, elastic, and finally solid. The odour is rather strong, unpleasant, and similar to that of our commercial galbanum.—*Central Blatt,* für 1852, No. xiii, *from Lond. Pharm. Jour. June,* 1852.

